# Targeting inflammation and immune activation to improve CTLA4-Ig-based modulation of transplant rejection

**DOI:** 10.3389/fimmu.2022.926648

**Published:** 2022-09-02

**Authors:** Marcos Iglesias, Daniel C. Brennan, Christian P. Larsen, Giorgio Raimondi

**Affiliations:** ^1^ Vascularized and Composite Allotransplantation (VCA) Laboratory, Department of Plastic and Reconstructive Surgery, Johns Hopkins University School of Medicine, Baltimore, MD, United States; ^2^ Division of Nephrology, Department of Medicine, Johns Hopkins University School of Medicine, Baltimore, MD, United States; ^3^ Department of Surgery, Emory University School of Medicine, Atlanta, GA, United States

**Keywords:** transplant rejection, costimulation-blockade, CTLA4-Ig, inflammation, immunological tolerance

## Abstract

For the last few decades, Calcineurin inhibitors (CNI)-based therapy has been the pillar of immunosuppression for prevention of organ transplant rejection. However, despite exerting effective control of acute rejection in the first year post-transplant, prolonged CNI use is associated with significant side effects and is not well suited for long term allograft survival. The implementation of Costimulation Blockade (CoB) therapies, based on the interruption of T cell costimulatory signals as strategy to control allo-responses, has proven potential for better management of transplant recipients compared to CNI-based therapies. The use of the biologic cytotoxic T-lymphocyte associated protein 4 (CTLA4)-Ig is the most successful approach to date in this arena. Following evaluation of the BENEFIT trials, Belatacept, a high-affinity version of CTLA4-Ig, has been FDA approved for use in kidney transplant recipients. Despite its benefits, the use of CTLA4-Ig as a monotherapy has proved to be insufficient to induce long-term allograft acceptance in several settings. Multiple studies have demonstrated that events that induce an acute inflammatory response with the consequent release of proinflammatory cytokines, and an abundance of allograft-reactive memory cells in the recipient, can prevent the induction of or break established immunomodulation induced with CoB regimens. This review highlights advances in our understanding of the factors and mechanisms that limit CoB regimens efficacy. We also discuss recent successes in experimentally designing complementary therapies that favor CTLA4-Ig effect, affording a better control of transplant rejection and supporting their clinical applicability.

## 1. Introduction

Long-term management of maintenance immunosuppression in organ transplantation remains complex. Calcineurin inhibitors (CNI)-based therapy, despite greatly decreasing acute rejection rates and improving 1-year outcomes, has not had as great effect on long-term allograft survival, and prolonged treatment is also associated with high risk of acute and chronic nephrotoxicity, post-transplant diabetes, elevated blood pressure, hyperlipidemia and neurotoxicity (1). Decades of research have shown that interfering with T cell costimulatory signals as a strategy to control undesired immune allo-responses, implementing the so called costimulation blockade (CoB)-based therapies, has the potential to induce tolerance to allogenic tissues and alleviate many of the unwanted side effects associated with current immunosuppressive therapies (2, 3). The only approach that has reached the clinic to date has been the use of cytotoxic T-lymphocyte associated protein 4 (CTLA4)-Ig, fusion protein that prevents the interaction of CD80 and CD86 on antigen presenting cells with CD28 on T cells. TCR stimulation without concomitant signaling through CD28 results in an abortive T cell activation and actuation of a program of apoptosis, induction of T cell anergy, and conversion into regulatory T cells (Treg) (4, 5).

Belatacept, a high-affinity version of CTLA4-Ig (6), was approved by the US Food and Drug Administration (FDA) for use in kidney transplant recipients in 2011 after evaluating results from the phase 3 randomized BENEFIT trials (7, 8). Despite its calcineurin-sparing benefits, and consistent with mouse-models, CD28 blockade *via* CTLA4-Ig alone does not fully prevent T cell activation (9). The use of CTLA4-Ig as a monotherapy has proved to be insufficient to induce long-term allograft acceptance in several transplant settings (7, 8, 10). Additionally, concerns with its long-term administration have also been raised due to a possible impact on the homeostasis of Tregs (11, 12). Consequently, many investigations have been trying to identify the main factors limiting CTLA4-Ig efficiency and to design clinically relevant complementary regimens to achieve its full therapeutic potential and supporting its successful clinical application.

**Figure 1:**
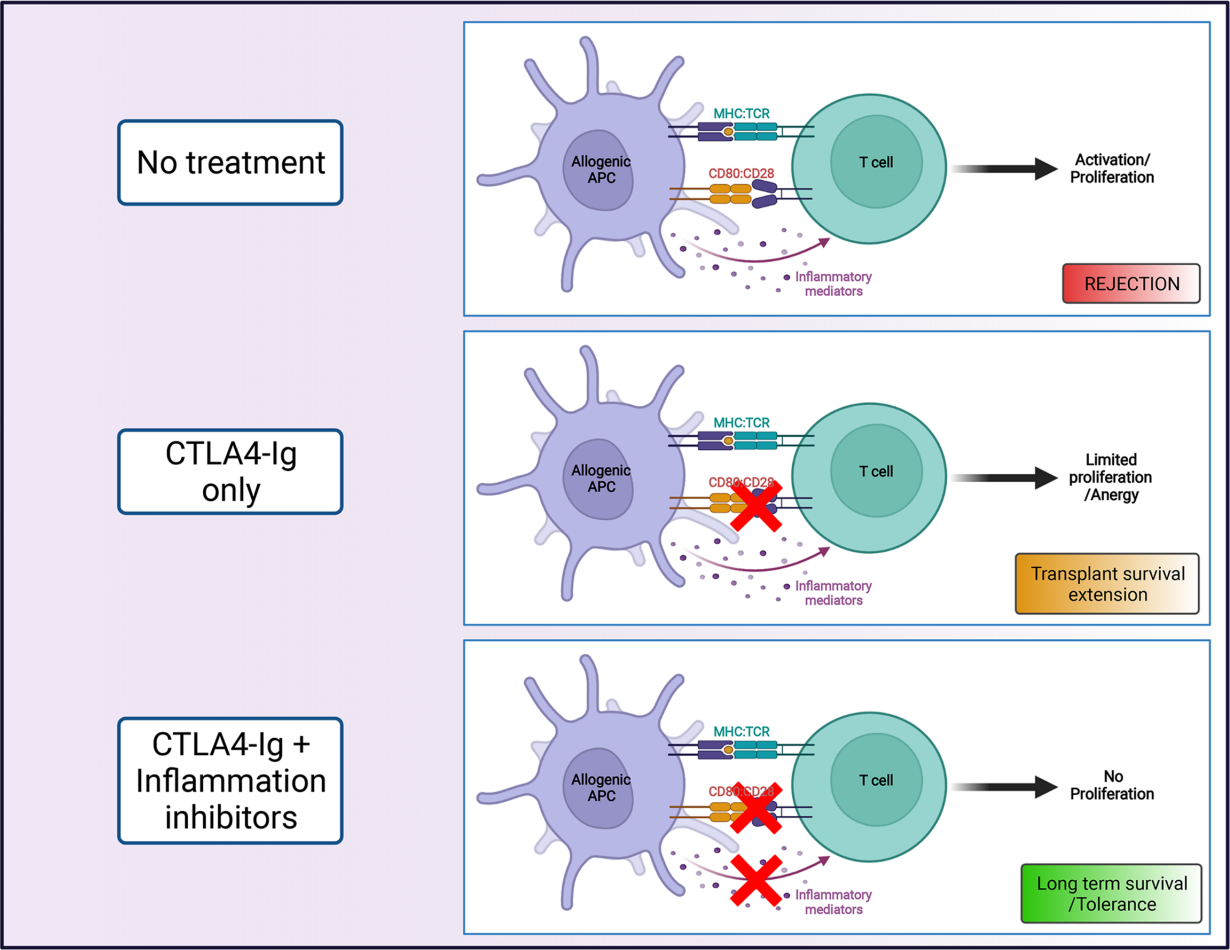
Costimulation blockade as strategy to promote transplant survival. The combination of CTLA4-Ig, that blocks interactions between CD28 and CD80/86 (signal 2), with agents that limit the contribution of inflammatory mediators (signal 3) on T cell activation, can promote long term transplant survival (Created with BioRender.com).

Multiple studies have demonstrated that events that induce an acute inflammatory response, with the consequent release of proinflammatory cytokines, can prevent the induction of or even break established immunological tolerance promoted *via* CoB regimens **(** **Figure 1** **).** Examples include the presence of damage-associated molecular patterns (DAMPs), pathogen-associated molecular patterns (PAMPs) (13–15) or infections (16, 17) at the time of transplantation; ischemia-reperfusion injury (IRI) induced by prolonged graft ischemia (18, 19); and the existence of graft-reactive memory cells induced by previous sensitization events (e.g. previous contacts with donor antigens – blood transfusions, previous transplants, pregnancies – or by heterologous immunity) (20, 21).

Fueled by the shortcomings of CTLA4-Ig, an important area of investigation has been the development of strategies to target additional costimulatory molecules to achieve lasting tolerance. The combined blockade of CD28 and CD154/CD40 signaling pathways was originally proposed as the strategy to achieve lasting graft survival in multiple rodent models of transplantation (9). However, a clinical trial with a humanized antibody to CD154 (the ligand of CD40) was halted due to thromboembolic events (22). Alternative clinically relevant costimulatory pathways (e.g. ICOS-ICOSL, OX40-OX40L, PD1-PDL1, TIGIT-CD155) are also being studied, however, the important advances achieved in this area will not be discussed here, as they have been comprehensively reviewed elsewhere (23).

**Figure 2:**
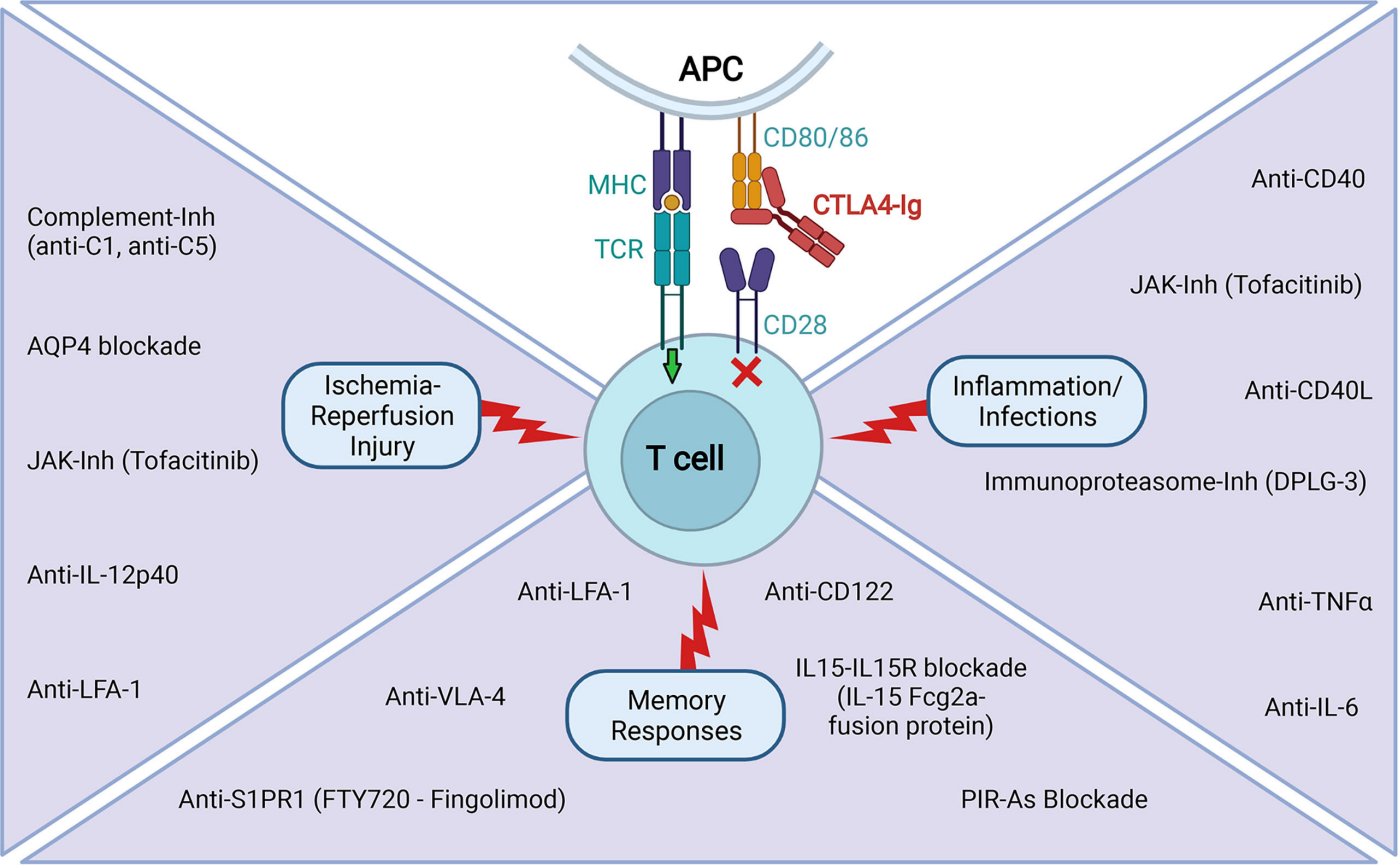
Tested experimental strategies to improve CTLA4-Ig efficacy in extending transplant survival. The reported use of agents that limit the contribution of signal 3 on T cell activation in situations of IRI, inflammation and infections, and pre-existence of memory responses that has proven to be efficacious in combination with CTLA4-Ig in extending transplant survival in various experimental settings (Created with BioRender.com).

This review highlights advances in our understanding of the factors (other than costimulatory molecules) that limit the efficacy of CTLA4-Ig-based regimens and discusses recent successes in experimentally designing complementary therapies that unleash CTLA4-Ig full potential, rendering a better control of transplant rejection and supporting their clinical applicability (summarized in **Figure 2** and **Table 1** **).** Moreover, we introduce novel areas of investigation, and we contextualize how targeting the signaling of inflammatory cytokines *via* JAK inhibitors could provide a significant advantage.

**Table 1:** List of tested experimental strategies to improve CTLA4-Ig efficacy in extending transplant survival.

Combined therapy	Transplant model	Molecular target
**Inflammation inhibitors**	Heart, skin (rodents) Islets, skin (rodents, NHP) Heart (rodents) Heart, islets (rodents) Heart (rodents)	Anti-CD40L (9) Anti-CD40 (24, 25) Immunoproteasome (DPLG-3) inhibitor (26) Anti-IL6 or/and anti-TNFα (27, 28) JAK Inhibitors (Tofacitinib) (29)
**IRI inhibitors**	Heart (rodents) Heart (rodents) Heart (rodents) Heart (rodents) Heart (rodents)	Complement inhibitors (C1, C5 inhibitors) (19, 30) Aquaporin 4 (AQP4) blockade (31) Anti LFA-1 (18, 32) Anti-IL-12p40 (33) JAK Inhibitors (Tofacitinib) (29)
**Control memory responses**	Islets (rodents) Skin (rodents), kidney (NHP) Heart (rodents) Skin (rodents) Heart (rodents)	IL-15 mutant/Fcy2a fusion protein (blockade IL15/IL15R pathway) (34, 35) Anti-CD122 (IL-2 and IL-15 receptor shared beta-chain) (36) Anti-S1PR1 (FTY720 – Fingolimod) (37) Anti LFA-1, anti-VLA-4 (20) PIR-As blockade (38)

## 2. Current landscape of CTLA4-Ig use in combination with other immunosuppressants

The combination of Belatacept with other immunosuppressants has shown benefits in animal models and paved the way to the initiation of clinical trials testing these protocols for clinical applicability (39, 40).

In kidney transplantation, only one calcineurin inhibitor–free regimen, *de novo* Belatacept in combination with mycophenolate and corticosteroids, is currently FDA approved for use in adult recipients who are seropositive for Epstein–Barr virus. Results have shown this regimen preserves renal function and has a minimal adverse effects profile (41, 42). However, the occurrence of high rates of acute rejection episodes, usually appearing early in the post-transplantation period, has prevented its widespread implementation, and utilization remains below 5% in the US (43). Several studies are evaluating the conversion to Belatacept post-transplant to avoid prolonged CNI exposure while decreasing the risk of acute rejection (44–46). Other investigations suggest that the optimal *de novo* Belatacept-based regimen may include lymphocyte-depleting induction in combination with an mTOR inhibitor (or Tacrolimus), instead of mycophenolate with or without corticosteroids (47–50).

The use of Belatacept for liver transplantation in the clinic has provided mixed evidence of efficacy. In a phase II exploratory trial of *de-novo* utilization of Belatacept with MMF, results failed to demonstrate safety or effectiveness, so its use was not recommended (51). Moreover, a follow up study attempting to maintain the same patients on MMF monotherapy after Belatacept withdrawal indicated lack of operational tolerance, as patients experienced graft dysfunction following the switch (52). Other studies showed some benefits with Belatacept conversion as method for CNI withdrawal (53), or using Belatacept with MMF as bridge to CNI therapy (54).

Belatacept is also a potential alternative to CNI in cardiothoracic transplantation (55). Two retrospective studies in lung and heart transplantation patients that had switched from CNI to Belatacept, as an alternative immunosuppression regimen post-transplantation, reported recovery in renal function and an acceptable safety profile (56, 57). More extensive investigations and clinical trials are however needed to obtain the necessary evidence to support this indication. In a rodent study, a short-course CTLA4-Ig-based conditioning regimen, which also included low-dose total body irradiation, bone marrow infusion, tacrolimus and antilymphocyte serum, favored the achievement of mixed chimerism, which correlated with the induction of long-term heart transplant survival and tolerance of secondary donor-specific skin grafts (58).

Experimental data in rodent skin allograft models has also been reported. A combination regimen of CTLA4-Ig with sirolimus or cyclosporine increased effectiveness in an experimental fully MHC-mismatched model of skin transplantation (59). In the same settings, combination with antilymphocyte serum with or without donor-specific bone marrow did not add any benefit to CoB monotherapy. Another report showed that the simple combination of CTLA4-Ig with low-dose anti-murine thymocyte globulin promoted engraftment by promoting Tregs in a context of limited alloreactive effector response (60).

Belatacept-based immunosuppression is also being investigated in the field of Vascularized Composite Allotransplantation (VCA), as extensively discussed in a recent review (61). Supported by the positive outcome of multiple preclinical models, conversion to Beletacept-based immunosuppression (in combination with MMF +/- low dose CNI) has been successfully attempted in a small number of VCA patients. *De novo* use of Belatacept, with MMF, steroids, and tacrolimus, is also being tested, but more data will need to be accrued to properly evaluate the applicability of such a strategy in VCA.

Overall, despite promising benefits, the implementation of Belatacept-based therapies in the clinic remains largely restricted to kidney transplantation. This landscape highlights the need for the identification and optimization of combination strategies with improved therapeutic efficacy that would favor a broader utilization for management of transplanted patients in multiple clinical settings.

## 3. Factors that affect CTLA4-Ig efficacy and related targeting approaches

### 3.1 Inflammation

Multiple preclinical studies have demonstrated that acute inflammatory events can prevent the induction of or even break established tolerance induced *via* CoB regimens (13–17, 62). These events range from surgical injury, infections, and ischemia reperfusion injury that initiate cellular cascades of alarm signals culminating in the production of pro-inflammatory agents. The impact of ischemia reperfusion injury on alloreactivity involves additional unique pathways and will be described in a dedicated sub-section.

Inflammatory cytokines such as type I interferons, IL-1, IFN-γ, IL-6, TNFα provide alternative signals that counteract the inhibition of conventional costimulation. As aforementioned, the combination of CTLA4-Ig with the blockade of CD40 signaling pathway, successfully extended transplant survival in multiple experimental animal models (9). In this context, extension of transplant survival is achieved in part due to the inhibition of secretion of inflammatory cytokines by antigen-presenting cells (APCs) caused by CD40/CD154 blockade (63). Following the interruption of clinical trials due to severe complications (22), multiple groups are currently working on improved versions of the anti-CD40L antibody to avoid the observed side effects (64–66). Alternatively, the blockade of CD40/CD154 pathway with anti-CD40 antibodies (Chi2020, 7E1-G2b), in combination with CTLA4-Ig, showed effects similar to the use of anti-CD154, inducing long term survival in a non-human primate (NHP) model of islet transplantation (Chi2020), and extending the survival of fully mismatched murine skin transplants (7E1-G2b) (24, 25). However, there are potential limitations in focusing specifically on the CD40 signaling pathway. A recent report suggested that a commonly ignored secondary receptor of CD154, CD11b, is important in favoring the accumulation of graft-infiltrating CD8 T cells after transplantation, independently from the engagement of CD40 (67). This observation explains why blocking CD40 instead of CD154 has been described as less effective for the inhibition of allograft rejection in some scenarios and supports the ongoing efforts to optimize anti-CD154 therapies.

Targeting the immunoproteasome (i-20S), a constitutive isoform of the proteasome highly expressed in T cells, dendritic cells, and B cells, has been explored to control inflammatory diseases like autoimmunity and alloimmunity. This approach would minimize the toxicity associated to the use of non-selective proteasome inhibitors (bortezomib, carfilzomib, ixazomib) which, despite providing good results in inflammatory and autoimmune diseases, can cause general immunosuppression (68). The use of DPLG3 (small molecule inhibitor of the i-20S b5i subunit) for a brief period after transplantation, suppressed cytokine release from blood mononuclear cells and the activation of dendritic cells (DCs) and T cells (26). It also diminished accumulation of effector T cells, promoted expression of exhaustion and coinhibitory markers on T cells, and synergized with CTLA4-Ig to promote long-term acceptance of cardiac allografts across a major histocompatibility barrier.

As above-mentioned, specific inflammatory mediators have been discovered that limit the efficacy of CTLA4-Ig monotherapy regimens. Different reports pointed to IL-1, IL-6, and TNFα as leading proinflammatory cytokines responsible for the anti-tolerogenic effects (69–71). IL-6 can impair the function of Tregs (key to the efficacy of CoB) by promoting proliferation of effector cells (72). The prolongation of transplant survival after CTLA4-Ig administration in recipients lacking this cytokine (IL-6-KO) suggests that the blockade of IL-6, or its signaling pathway, has a synergistic effect with strategies that inhibit T_H_1 responses, ultimately promoting long-term allograft survival (70). In line with this observation, treatment with an anti-IL-6 antibody promoted allogenic bone marrow engraftment and prolonged graft survival in an irradiation free murine transplantation model receiving CoB, a result associated with expansion of endogenous Tregs and inhibition of DCs and memory CD8 T cells (27). TNFα also plays an important role in immune regulation (73). It has been found responsible for an increase in T cell allogenic responses (74) and the impairment of peripheral tolerance induction to allogenic pancreatic islets (69). A synergistic role for IL-6 and TNFα acting together to promote T cell alloimmune responses and impairing the ability of Treg cells to suppress effector T cell alloimmunity has also been described (28). Thus, accumulating evidence suggests the need to combine CoB therapies with inhibition of either the production or the signaling of multiple inflammatory cytokines to achieve a robust modulation of transplant rejection.

The identification of Janus kinases (JAKs), non-receptor tyrosine kinases, as critical components of the signaling pathway of multiple proinflammatory cytokines (e.g. type 1 interferons, IFN-γ, IL-6), made them an attractive drug target to simultaneously inhibit the effect of several inflammatory mediators. Initial efforts lead to the development of JAK inhibitors (JAK-Inh), small molecules that interfere with JAK enzymatic activity (75). JAK-Inh also appeared as a more cost-effective and efficient alternative to biologics to target cytokines, as not all patients respond adequately to biologics-based treatments (76). First generation JAK-Inh were initially tested in transplantation as immunosuppressants and compared with standard of care. In randomized clinical trials in kidney transplantation the use of a fixed dose of tofacitinib, a pan-JAK-Inh currently FDA approved for the treatment of rheumatoid arthritis, psoriatic arthritis, and ulcerative colitis, in combination with mycophenolic acid and corticosteroids, demonstrated a lower incidence of rejection and better renal function than cyclosporine A. However, the safety profile was poor, showing an increased risk of infection and malignancy when compared to CNI-based regimens (77–79). Unfortunately, after these results, the investigation of tofacitinib as a maintenance immunosuppressant in transplantation was halted. Interestingly, revised analysis of the data from these studies and the results of a long-term extension trial, all highlighted the importance of controlling tofacitinib dosing (probably used at too high of a dose in the initial trials) and the need for careful evaluation of the drug combination strategy implemented. It was suggested that close monitoring of the therapeutic dose and targeting reduced exposure over time post-transplant, may improve outcomes and reduce side effects (80). Encouraged by the lessons learned from these reports, the use of tofacitinib as part of a very different combination strategy together with CTLA4-Ig has started to be explored. Using a mouse model of heart transplantation, the combination of CTLA4-Ig with a short period of daily tofacitinib administration revealed a profound synergistic effect that promoted long-term transplant survival (29). The protective effect of the combination of CTLA4-Ig and tofacitinib was associated with inhibition of the maturation of APCs, inhibition of the differentiation of effector T cells, and promotion of intra-graft accumulation of Tregs. This study demonstrated the powerful synergism between CTLA4-Ig and JAKs-inhibition, and suggests their combined use is a promising strategy for improved management of transplanted patients that should be further investigated.

### 3.2 Ischemia reperfusion injury

Ischemia/reperfusion injury (IRI) is a universal consequence of solid organ transplantation, initiated after interruption of blood supply following organ procurement. In renal transplantation it is currently one of the most prominent causes of delayed graft function (especially for deceased donors), in part due to the use of new allocation systems and novel approaches that have introduced an extension in organ preservation time (81, 82). The negative impact of IRI on cells and tissues arise from the complex network of events including oxygen and nutrient deprivation, disruption of cellular homeostasis, with a switch from aerobic to anaerobic metabolism and resulting in cellular acidification, accumulation of reactive oxygen species and inflammation. An increased release of proinflammatory mediators, cytokines, chemokines, and expression of adhesion molecules are induced by IRI and contribute to ischemic tissue damage resulting in the release of DAMPs (83, 84). The combination of these effects promotes, directly or indirectly, enhanced anti-donor cellular and humoral responses. Prolonged cold ischemia storage is considered an independent risk factor for poor transplant outcome, having been associated with a higher incidence of delayed graft function, acute and chronic rejection (85). It is also considered one of the leading causes of failure of prolongation of transplant survival by CTLA4-Ig due to its promotion of “costimulation independent” activation of anti-donor responses (18, 19). Different approaches are being investigated to minimize the effects of IRI.

Several studies have revealed that many of the pathological effects of IRI are complement dependent (86). Targeting the complement cascade, which is an important component of the innate immune system, has minimized graft injury initiated by donor reactive antibodies and limited vascular allograft rejection in sensitized recipients (87, 88). Building on this understanding (86), and that alternative complement pathway components contribute to T cell activation and differentiation, inhibition of complement activity has been investigated as an immunomodulatory strategy in transplant settings (89, 90). The inhibition of C5 limited graft injury in human kidney transplant recipients (88). Targeting C5 also had synergistic effect with CTLA4-Ig prolonging survival of murine heart allografts subjected to IRI (30). Mechanistically, the use of anti-C5 mAb prevented the formation of C5a and C5b, resulting in limited induction of T_H_1 alloreactive cells and inhibition of primary responses to donor antigens (30). The same group identified an important role for the mannose-binding lectin (MBL) complement pathway (but not the alternative pathway), in the deleterious effect of IRI. CTLA4-Ig treatment of C3^-/-^ recipients of ischemic heart allografts, as well as the use of mbl1^-/-^mbl2^-/-^ transplant recipients, prolonged survival compared to wild type recipient mice. This prolongation was associated with profound inhibition of the production of pro-inflammatory cytokines and a limited presence of intra-graft alloreactive activated T cells. Importantly, this group showed a benefit with the use of the FDA approved complement inhibitor C1-INH, which targets the MBL pathway, in combination with CoB. In a murine heart transplantation model, C1-INH was particularly effective when administered within the first 24h post-transplant of ischemic allografts, supporting the need for further studies to test the clinical applicability of this regimen (19).

Promising results were also obtained in experimental transplantation when targeting the Aquaporin 4 (AQP4) signaling pathway. Aquaporins are a family of water channels that facilitate homeostasis but are also involved in modulation of tissue injury and inflammation. AQP4 deficiency results in reduced myocardial tissue damage during infarct and IRI (91). A recent report in a murine model showed encouraging results for the control of IRI in transplantation when AQP4 was blocked during donor allograft collection and storage and short time after transplantation (31). The combined administration of an AQP4 inhibitor and CTLA4-Ig synergistically prolonged the survival of heart allografts. *In vitro* observations of a reduced T cell proliferation and cytokine production following AQP4 blockade, suggest that these effects could help limit the anti-graft response post-ischemia and hence favoring transplant survival (31).

IRI is responsible for the increase in the early infiltration of innate and adaptive leucocytes into the allografts. Neutrophils and macrophages quickly migrate to the ischemic area sensing DAMPs, become activated and lead to a release of chemokines and cytokines (92). Macrophages increase their processing and presentation of allo-antigens, contributing to adaptive immune activation, enhancing the effector functions displayed by early infiltrating memory CD8 T cells, and favoring transplant rejection (18). The simple prevention of early migration of donor specific memory CD8 T cells to the allograft by the administration of anti- leukocyte function associated antigen-1 (LFA-1), an integrin involved in adhesion, activation and trafficking of leukocytes, favored the survival of the transplant (18, 32). In this context, it is plausible to consider an involvement of the recently described “virtual memory” T cells, a population of memory CD4 T cells displaying a T_H_1-like phenotype that is generated at the steady state from naïve T cells, in the absence of foreign antigen recognition, and that can be reactivated in a TCR-independent way by IL-12. This population is considered a contributor to enhanced responses in autoimmune and inflammatory diseases (93, 94). With prolonged cold ischemia, endogenous memory CD4 T cells stimulate graft-infiltrating dendritic cells to produce homodimers of IL-12-p40 (IL-12 subunit) that in turn activate anti-graft memory CD8 T cells (33). This process contributes to CTLA4-Ig-resistant allograft rejection observed in a murine model of heart transplantation. The combined therapy of CTLA4-Ig with anti-p40 antibodies proved to be efficacious at extending transplant survival in the mentioned IRI conditions (33), suggesting the possible use of this strategy to counter CTLA4-Ig-resistant allograft rejection mediated by memory CD8 T cells.

The use of JAK-Inh, already mentioned in the previous section, has also proved to be beneficial to minimize the inflammation and proinflammatory cytokine release characteristic of IRI in a mouse model. Combination of short course of tofacitinib with CTLA4-Ig was able to extend survival of heart allografts subjected to 4h cold ischemia, settings where CTLA4-Ig monotherapy is unable to delay graft rejection. Long-term survival achieved with this combined therapy was associated to a decrease in effector T cell allo-response, and correlated with *in vitro* observations of a full control of T cell proliferation when T cells were stimulated in the presence of both inhibitors, even if exposed to proinflammatory conditions (29). This report highlights the versatility of combining CTLA4-Ig with JAKs-inhibition for improving immunoregulation while limiting the negative effect of inflammatory mediators and indicate the need for additional studies to prove its clinical potential for improving management of transplanted patients.

### 3.3 Pre-existing memory alloreactivity (sensitization)

One of the biggest barriers to achieving effective modulation of rejection (and possibly allograft tolerance) is the presence of immunological memory toward donor antigens in the recipient before transplantation. Memory cells can develop due to previous sensitization events (e.g. previous contacts with donor antigens through blood transfusions, previous transplants, pregnancies, and by development of heterologous immunity) (20, 21, 95) or *via* homeostatic proliferation while recipients are under immunosuppression (96). Memory responses are faster and more robust, in most cases translating into rejection being more resistant to pharmacologic immunosuppression (97–99). The difficulties presented by pre-existing anti-donor memory apply to CoB regimens too. Memory CD4 and CD8 T cells are less dependent on costimulation for activation, and they can mount a quick allo-response, promote inflammation, and attack and destroy the transplant despite application of CoB treatment. Combination therapies aiming to overcome CTLA4-Ig resistant rejection mediated by memory lymphocytes are being actively investigated.

In humans, different reports indicated that pre-transplant accumulation of different T cell memory subsets are associated with increased risk of CoB resistant rejection. These subsets encompass: a CD28+CD4+ effector memory T cells (100), antigen-experienced CD57+PD1-CD4 T cells (101–103) and a subset of memory CD8 T cells that lacks expression of CD28 (104). IL-15 is a powerful T cell growth factor with particular importance for the maintenance and proliferation of memory CD8 T cells (105, 106). The CD28-CD8 T cell subset, in both non-human primates and humans, relies on cytokines like IL-15 and IL-2 for activation. CoB cannot act to directly decrease IL-15 expression because epithelial and endothelial cells, and macrophages, not T cells, are the primary cellular sources of this cytokine (107). IL-15 was found to be responsible for the induction of CTLA4-Ig resistant proliferation of alloreactive memory CD8 T cells from renal transplant patients (104). In settings where CoB-resistant rejection could be mediated by memory CD8 T cells, targeting IL-15/IL-15R pathway in combination with CTLA4-Ig represents a potent strategy for the induction of transplant tolerance. The use of an IL-15 antagonist, IL-15 mutant/Fcy2a (a fusion protein that specifically binds to IL-15Rα, but not to the common γ-chain, shared with other cytokines like IL-2) in combination with CTLA4-Ig, favored the achievement of transplant tolerance in a rodent semi-allogenic islet transplantation system (108). When tested in a fully MHC-mismatched scenario, the combined therapy also extended allograft survival further than CTLA4-Ig alone (34, 35). Additional studies targeting CD122 (IL-15 receptor-β-chain, shared with IL-2) also showed promising results with a synergistic protective effect when combined with CTLA4-Ig (36). The treatment abrogated both primary and memory CD8 T cell responses to transplanted tissues, in mice and non-human primate models. Mechanistic studies dissecting the effect of the anti-CD122 blocking antibody supported a role for IL-15 in memory T cell activation, while the prevention of primary allo-specific responses was more likely due to the blockade of IL-2 signaling (36).

The blockade of migration of memory cells to the graft has also been investigated in combination with CTLA4-Ig. A modification or restriction of lymphocyte homing receptors is a proposed strategy to promote transplant survival. The sphingosine 1-phosphate receptor-1 (S1PR1) functional antagonist FTY720 (FDA approved as Fingolimod), inhibits lymphocyte egress from thymus and lymph nodes (109) and more recently has been described to also hinder DC migration to lymph nodes and their secretion of IL-12 and IL-23 (110). In an experimental murine model of BALB/c.2W.OVA donor heart transplantation into pre-sensitized recipients, the combination of CTLA4-Ig with FTY720 limited anti-donor IFN-γ responses (already achieved with CTLA4-Ig monotherapy), inhibited alloantibody production, and restrained T cell recruitment to the graft, with a consequent extension of transplant survival (37). The levels of donor-reactive effector memory T cells after treatment were lower than pre-transplant, and the authors speculated that the combined therapy might also potentially serve as a T-cell desensitizing protocol (18, 32). Another approach to interfere with memory T cells migration is the use of integrin antagonists. In a skin allograft model where traceable OVA specific CD8 T cells responded to OVA-expressing donor skin, the resistance of memory T cells to CoB consisting of CTLA4-Ig and anti-CD40L, was abrogated when this regimen was coupled with either anti-VLA-4 or anti-LFA-1 (20). Mechanistic studies revealed that in the presence of CoB, anti-VLA-4 impaired T cell trafficking to the graft but not memory T cell recall effector function, whereas anti-LFA-1 attenuated both trafficking and memory recall effector function. As antagonists against these integrins are already clinically approved (anti-LFA-1, Efalizumab, was approved in 2003, however later suspended in 2009 due to several patients developing progressive multifocal leukoencephalopathy) (111, 112), these findings may have significant translational potential for future clinical transplant trials to minimize CoB resistance associated to the presence of memory CD8 T cells (20).

Initially thought to be confined to T and B lymphocytes, it is now evident that innate myeloid cells can also acquire temporary features of immunological memory, contributing to the amplification of the effects of inflammation and infections after an initial insult (113). This phenomenon defined as “trained immunity” is orchestrated by epigenetic changes (and not by permanent genetic mutations or reprogramming) and confers myeloid, NK cells, and innate lymphoid cells an increased responsiveness to secondary stimuli recognized through pattern recognition receptors (113). In the setting of transplantation, innate myeloid cells such as monocytes and macrophages are able to retain a temporary memory to prior challenges through MHC-I receptors called PIR-As (A-type paired immunoglobulin-like receptors) (38). Ly6C^hi^ monocyte/dendritic cells and macrophages activated in a primary allo-response, mount a greater inflammatory reaction after a second challenge with the same non-self MHC complex, contributing to accelerated transplant rejection. The contribution of innate immunity to transplant rejection has been confirmed in murine models of kidney and heart transplantation with studies using PIR-A blocking antibodies or Pira^-/-^ recipients, showing attenuated responses to donor antigens. The combination of PIR-As blockade with CTLA4-Ig treatment resulted in a synergistic effect, preventing both acute and chronic rejection in a murine model of heart transplantation (38). These recent results indicate that innate memory is another important player in counteracting the therapeutic efficacy of CTLA4-Ig and they suggest a new line of investigation for the development of intervention strategies to improve transplant outcomes.

## 4. Future outlook

### 4.1 Belatacept and Tregs

The role of Tregs in the induction and maintenance of tolerance is well-recognized and there are major ongoing efforts to realize Treg-based clinical applications to promote long-term organ allograft survival (114–116). In addition to directly controlling proliferation of T and B cells, Treg also suppress immune responses and decrease inflammation by limiting the maturation of DCs – ultimately resulting in a more tolerogenic phenotype of these cells, characterized by decreased secretion of proinflammatory mediators and reduced expression of costimulatory molecules (117, 118). Encouraged by positive safety results obtained in phase I trials (119, 120), multiple clinical trials are underway worldwide to test the efficacy of Treg adoptive cell therapy to improve the management of transplanted patients (121). Importantly though, animal models clearly indicate that Tregs are not capable of inducing transplant tolerance when used as single agent (122, 123). In this regard, the possibility of combining CTLA4-Ig with Tregs in transplantation is appealing, but also controversial. As CD28 is required for Treg generation and CTLA4 is essential for Treg function, blockade of the interaction of both molecules with their ligands CD80 and CD86 on APCs by CTLA4-Ig may be detrimental to Treg survival and function. This potential issue is exemplified by the observation that CTLA4-Ig administration accelerated rejection in a single MHC class II-mismatched mouse model of heart transplantation, where the naturally occurring long-term allograft survival is dependent on Tregs (11). Similarly, in another model of single MHC class II-mismatched skin transplantation, where the expansion of endogenous Tregs extends graft survival, CTLA4-Ig reduced Treg-dependent immunomodulation and restored T_H_1 alloreactivity (12). More recent studies suggest, however, that the counterproductive effects of CD28 blockade on Treg can be avoided by refinement of the dose and timing of CTLA4-Ig administration (124, 125). For example, the adoptive transfer of recipient Tregs obviates the need for cytoreductive conditioning (i.e. irradiation or cytotoxic drugs) in a fully allogeneic bone marrow transplantation model when given together with rapamycin and CoB (anti-CD40L and CTLA4-Ig). This regimen induced durable mixed chimerism and tolerance to skin and heart allografts (126, 127), and its clinical applicability is currently being assessed in an ongoing clinical trial (128). In addition to therapies that involve the transfer of ex-vivo expanded Tregs (polyclonal or Ag-specific using TCR-gene transfer and chimeric antigen receptor technology) to increase the Treg pool (129, 130), other approaches that aim to directly expand Treg *in vivo* are being investigated. These approaches are based on the administration of IL-2 (essential cytokine for Treg expansion), in a concentration or form that is biased for a more selective Treg engagement. Examples are the use of IL-2 mutants (muteins) (131, 132) and IL-2/Anti-IL-2 immuno-complexes (133, 134), all of which are designed to increase cytokine half-life and to skew binding toward Treg cells (expressing the high affinity IL-2 receptor subunit CD25). The combination of these therapies with CoB is a promising strategy to circumvent the possible deleterious effect of CTLA4-Ig and maximize its therapeutic efficacy.

### 4.2 Metabolic inhibitors

The growing field of immunometabolism has shown that metabolic reactions are not only used for the cells to generate energy to perform their functions, but they are also a way to control immunity and inflammation (135–137). The discovery that T cells have different metabolic requirements depending on their activation status (proliferation, differentiation, effector function) or cell subtype, has opened a new avenue of investigation to learn how to control cell responses by targeting their metabolism. Initial studies in the oncology field have shown that activated T cells markedly upregulate glycolysis even in the presence of oxygen to satisfy the energetic demand of this process, while naïve and Treg cells rely on more conventional processes such as oxidative phosphorylation and fatty acid oxidation (138). Studies in a rodent transplant model demonstrated the immunosuppressive effect of continuous anti-metabolic therapy targeting glycolysis and glutamine metabolism (via administration of the glucose analog 2-Deoxy-d-Glucose, the glutamine analog 6-diazo-5-oxo-L-norleucine, and metformin), resulting in extension of allograft survival through the inhibition of effector cells and the induction of Treg (139). The need for continuous treatment, however, indicated that this anti-metabolic strategy did not promote the actuation of tolerogenic mechanisms capable of sustaining transplant tolerance. However, when metabolic inhibition was paired with CTLA4-Ig, this led to enhanced skin allograft survival and promoted long-term heart transplants acceptance in the absence of maintenance treatment (140). Further investigations of the metabolic demands of immune cells during rejection, together with the identification of more selective inhibitors, has the potential to define a novel therapeutic strategy that can safely and effectively synergize with CoB in the induction of transplant tolerance.

### 4.3 The application of next generation JAK-Inh

First generation JAK-Inh have been under investigation as therapeutics to control the deleterious effect of proinflammatory mediators in inflammatory and autoimmune diseases, as well as transplantation. Following positive results of the REACH-2 clinical trial in graft-versus-host disease (GVHD), Ruxolitinib, a JAK1/2 inhibitor, obtained FDA approval, and it is now considered the gold standard in glucocorticoid-refractory acute GVHD treatment (141). As aforementioned, a clinical trial of tofacitinib as immunosuppressant in kidney transplantation showed a lower incidence of rejection and better renal function when compared to a cyclosporine-based regimen, but the poor safety profile observed at the doses tested (with a probable contribution by the combination with the anti-metabolite agent MMF) halted its further clinical investigation (77–79). Additional analysis of data gathered from clinical trials informed that with the proper dosing and careful evaluation of the drug combination employed, the utilization of this inhibitor could provide beneficial results in the transplant field (80). In fact, as discussed in a previous section, the experimental use of a short-course tofacitinib treatment in combination with CTLA4-Ig, demonstrated a synergistic effect extending survival of heart allografts (29). In concordance with other results (142, 143), exposure of DCs to tofacitinib not only reduced the upregulation of costimulatory molecules (by interfering with the JAK/STAT signaling pathway intrinsic to maturation), but it also limited the secretion of factors, like IL-1 and TNFα, that are involved in CoB-resistant transplant rejection (29). However, the impact of JAK-Inh on the homeostasis and function of Tregs needs to be also considered. Long-term administration of JAK-Inh has been associated with a decrease in Treg abundance in multiple settings (144–146). However, other reports indicate that a shorter course does not have a negative impact on this population and that in all cases, the suppressive function of the remaining Treg population is unaffected by JAK-Inh (29, 147, 148). These results clearly suggest the need to further investigate (experimentally and clinically) the utilization of JAK-Inh for the control of multiple inflammatory responses and to support the efficacy of CoB regimens in organ transplantation.

First generation JAK-Inh block all three JAKs and consequently inhibit the action of a number of cytokines on multiple cell types, unfortunately causing significant side effects with their prolonged use. Their promising therapeutic effects sparked great interest in the generation of more selective JAK-Inh, aiming to maintain efficacy while reducing adverse effects (especially those resulting from JAK2 inhibition). Current studies are investigating second-generation JAK-Inh for GVHD (149), several of which have already been FDA approved for the treatment of autoimmune diseases (E.g. Baricitinib, JAK1/2-inh, for RA (150); Upadacitinib, JAK1-Inh, for RA (151); and the not FDA approved Filgotinib, JAK1-Inh (152)) (153). These inhibitors have higher specificity and could present themselves as improved alternative to ruxolitinib or tofacitinib to pair with CTLA4-Ig for a more selective regulation of inflammation (154). From the overview presented herein of the factors that negatively affect CTLA4-Ig efficacy, it is noteworthy that most of the negative effect is directly or indirectly related to inflammatory or homeostatic cytokines, potential targets of modulation by JAK-Inh. It is reasonable to speculate that the use of JAK-Inh could become a valid alternative to more expensive biologics or other aforementioned therapies to maximize CTLA4-Ig efficacy in the development of therapies that will provide safe and effective management of transplanted patients in multiple clinical settings.

In September 2021, the FDA required an update on the prescribing information (a black box warning) for certain JAK-Inh in the treatment of chronic inflammatory conditions (155). This update came after revision of safety profile studies that determined an increased risk of serious side effects (heart attack, stroke, blood clot, cancer, and death) after continuous and prolonged JAK-Inh use. Considering this new regulation, the additional optimization of administration protocols appears paramount to improve their safety profile and expand their use to other inflammatory disorders and transplantation. The data in rodent models suggesting that the combination of CTLA4-Ig with a transient (rather than continuous/life-long) administration of a JAK-Inh enables proper control of transplant rejection indicates that alternative administration regimens are feasible and effective (29). Implementation of localized delivery systems is also an interesting strategy proven to reduce toxicity deriving from systemic drug exposure, allowing the design of safer strategies of management of transplanted patients. The use of biomimetic nanoparticle platforms and hydrogels are examples of these approaches (156–159). In this regard, a recent study looked at the possibility of limiting the systemic exposure to tofacitinib while maintaining synergism with CTLA4-Ig. Using a single administration of a novel injectable peptide-based hydrogel containing crystals of tofacitinib, the authors demonstrated in a rodent model that delivery of tofacitinib localized exclusively around the transplant preserved synergism with CTLA4-Ig in promoting long term graft survival (160).

## 5. Conclusions

The limited improvement in the long term management of transplanted patients of the past two decades calls for new and more effective treatment strategies (81). As summarized in this review, research of the past few years revealed novel understanding of the mechanisms of activation of the immune system that challenge old paradigms. These new observations highlight the important, and often unappreciated, role of inflammatory events in limiting the capacity to effectively control transplant rejection. There is then an exciting opportunity for targeting inflammatory perturbations in combination strategies, with realistic translational potential, that will provide better control of alloreactivity. Implementation of biologics, JAK-Inh, or other inhibitors of inflammation could represent the missing piece of this very important puzzle. However, we still need to fully understand the subtle connections to both beneficial and detrimental effects in the utilization of these agents. This notwithstanding, great optimism accompanies the ongoing effort to validate many of these new experimental protocols in large animal models as well as in testing their clinical scalability and efficacy in human transplantation.

## Author contributions

MI and GR drafted and reviewed the manuscript. DB and CL reviewed the manuscript and provided insightful feedback. All authors contributed to the article and approved the submitted version.

## Funding

This work was supported in part by JDRF strategic research agreements 2-SRA-2016-304-S-B and 2-SRA-2016-310-S-B as well as United States Army Medical Research Acquisition Activity (USMRAA) grants W81XWH-18-1-0789 and W81XWH-19-1-0352 (all to G.R.). DCB is supported in part from the Melody and Raymond Ranelli Fund and from the Charles T. Bauer Charitable Foundation. CPL is supported in part by the James M Cox Foundation and the Carlos and Marguerite Mason Trust.

## Acknowledgments

We are deeply thankful to the Department of Plastic & Reconstructive Surgery at Johns Hopkins School of Medicine for administrative and secretarial support.

## Conflict of interest

GR is an inventor in pending patent applications on the localized delivery of small molecule inhibitors (including JAK-Inh). DCB receives research support from Allovir, Amplyx, CareDx, and Natera, he is consultant for CareDx, Hansa, Medeor, Sanofi, and Veloxis and receives honoraria from CareDx, Sanofi, and Veloxis. CPL is on the Scientific Advisory Board of CareDx and Eledon and receives clinical trial support from Bristol Myers Squibb.

The remaining author declare that the research was conducted in the absence of any commercial or financial relationships that could be construed as a potential conflict of interest.

## Publisher’s note

All claims expressed in this article are solely those of the authors and do not necessarily represent those of their affiliated organizations, or those of the publisher, the editors and the reviewers. Any product that may be evaluated in this article, or claim that may be made by its manufacturer, is not guaranteed or endorsed by the publisher.
